# Development and Application of a Quality Assessment Tool for Oncological Question Prompt Lists

**DOI:** 10.1007/s13187-023-02290-z

**Published:** 2023-03-30

**Authors:** Lena Josfeld, Nathalie Zieglowski, Julia Möller, Christian Keinki, Jutta Hübner

**Affiliations:** Department of Hematology and Medical Oncology, University Hospital Jena, Jena, Germany

**Keywords:** Question prompt lists, Patient information material, Quality assessment tool, Patient-centered care, Patient-physician communication

## Abstract

No specific quality criteria yet exist for question prompt lists (QPLs), so this study aims to develop a quality assessment tool to then use for an evaluation of online-available QPLs. An online search was conducted for German-language QPLs using different internet search engines and terms. A wide range of existing quality criteria for patient information was adapted to the field of QPLs to build an assessment tool and evaluate all identified QPLs by four independent raters. All new quality criteria were applicable to QPLs. The overall quality of 46 oncological QPLs was low, though the tool’s subcategories were mostly fulfilled to over 80% by at least one QPL. For-profit organizations published lesser quality than medical organizations. The quality of breast- and prostate-cancer-specific QPLs was higher than that of general ones. High-quality QPLs could be created if more aspects were taken into account, but the available QPLs only focus on few quality aspects. The ambiguous results of effectiveness studies to date may be a result of vastly differing quality of the QPLs used for the interventions. The criteria provided in this study present a solid basis to assess the quality of QPLs. The creation of future QPLs as well as effectiveness studies should be more firmly based on quality criteria.

**Supplementary Information:**

The online version contains supplementary material available at 10.1007/s13187-023-02290-z.

## Introduction

Oncological conditions and treatments are complex matters for patients to understand and make decisions about. However, in order to adhere to ethical and societal demands of a person’s self-determination [1], medical decision-making processes require patients’ active involvement. Shared decision-making, for which patients and physicians confer about different options and preferences to reach a decision, has become today’s widely accepted gold standard, despite it still facing various challenges in implementation, like concerns regarding time constraints and adverse effects, uncertainty about the decision, and—especially—communication difficulties between patient and physician [2]. While some patients prefer to have their trusted physicians make decisions for them [3], the demand for information and active involvement is generally high [3, 4]. Gathering information during a consultation can be a challenge for both patients and clinicians. While patients are often enough encouraged to ask questions or even to write them down before their next consultation, many are not sure which questions to ask and how [5]. They remain unaware of impending decisions, of important issues they need to know about, or even that a decision has been made [6].

Question prompt lists (QPLs) have become an increasingly available medium of potential support for patients in this situation. They list any number of questions that may be of potential interest to the patient, many focusing on enabling patients to make an informed decision about their health and treatment.

Research on the effectiveness of QPLs so far has shown ambiguous results, as several reviews demonstrate [7–10]. For example, while generally more questions are being asked or more different topics discussed when patients are given QPLs before a consultation, effects on consultation length are conflicting. Some studies find no effects, others a shortening of the consultation, others a longer consultation time. Some studies find positive effects on information recall, though possibly depending on other additional factors; other studies find no such effects. No effects on patients’ satisfaction with the consultation or with the decision could be found, but a self-generated question list may improve satisfaction with the consultation.

In summary, while research has been conducted on the effectiveness of QPLs, there is hardly any insight on what may be the core quality aspects for this type of communication tool. Most studies assess the effectiveness of a QPL that was specifically designed for their intervention, but detailed descriptions of the used material are rare [8]. This study attempts to create a first catalog of quality criteria specifically for QPLs. Such criteria may be used by researchers, patients, and clinical practitioners alike in order to identify good-quality material to facilitate patient-clinician communication in everyday practice. Especially patients who are looking for support before their consultation with a physician may come across all manner of QPLs, the quality of which often remains unknown. In the second step of this study, we apply the created quality criteria to our own online findings in order to gain an initial overview of the variety and quality of QPLs available to German-speaking cancer patients who look for information on the internet. To our knowledge, no such evaluation has been conducted to date.

## Materials and Methods

We conducted an extensive online search to identify QPLs in German language that are currently available online. A specifically designed assessment tool of quality criteria for QPLs was then used to evaluate the quality of those search results.

### Online Search

The online search was conducted from mid-February to mid-March 2021, using the following highly frequented search engines in Germany over the previous 6 months [11]: google.de (91.93% market share), bing.de (5.07%), and yahoo.com (0.61%). In order to determine the search terms, we analyzed the titles of QPLs published by established German health organizations and reviewed scientific literature to identify additional keywords. Included were general terms as well as specifically oncological ones, focusing on the two types of cancer with the highest case numbers in Germany according to the Robert Koch Institute: breast cancer for women (69,900 new cases in 2018 [12]) and prostate cancer for men (65,200 new cases in 2018 [13]). The final search terms were “list of questions for visiting the doctor” [German original: “Frageliste für den Arztbesuch”], “checklist for medical consultation” [“Checkliste Arztgespräch”], “questions to ask the doctor” [“Fragen an den Arzt”], “preparing medical consultation” [“Vorbereitung Arztgespräch”], “checklist cancer” [“Checkliste Krebs”], “checklist breast cancer” [“Checkliste Brustkrebs”], and “checklist prostate cancer” [“Checkliste Prostatakrebs”]. They were entered into the search engines without inverted commas based on the strategic deliberations that this would be less limiting to search results and that most patients would be likely to conduct online searches this way.

Every search term was applied to each of the three search engines successively; browser histories and cookie caches were emptied in between searches. The first 50 resulting URLs of each search were saved for review, yielding a total of 1050 URLs. Duplicates were removed before the content was analyzed according to the following inclusion and exclusion criteria:

Included were all QPLs in German language available without cost or previous registration. QPLs had to be accessible via a maximum of two clicks from the original URL and could be either viewed on the website directly or downloaded as a.PDF or.doc file. The content had to be in written form, contain a minimum of three questions that could be posed to a treating physician, and had to be tailored to the patient or another immediately affected person close to them (e.g., family/caregiver).

The count of the first fifty results excluded those listed as advertisement. Excluded were also malfunctioning links or downloads, websites requiring payment or registration to access content, question lists outside medical context, question lists tailored to clinicians instead of patients, and lists of questions that were already answered and thus focused on providing direct information rather than assisting with acquiring them from the treating physician.

**Fig. 1:**
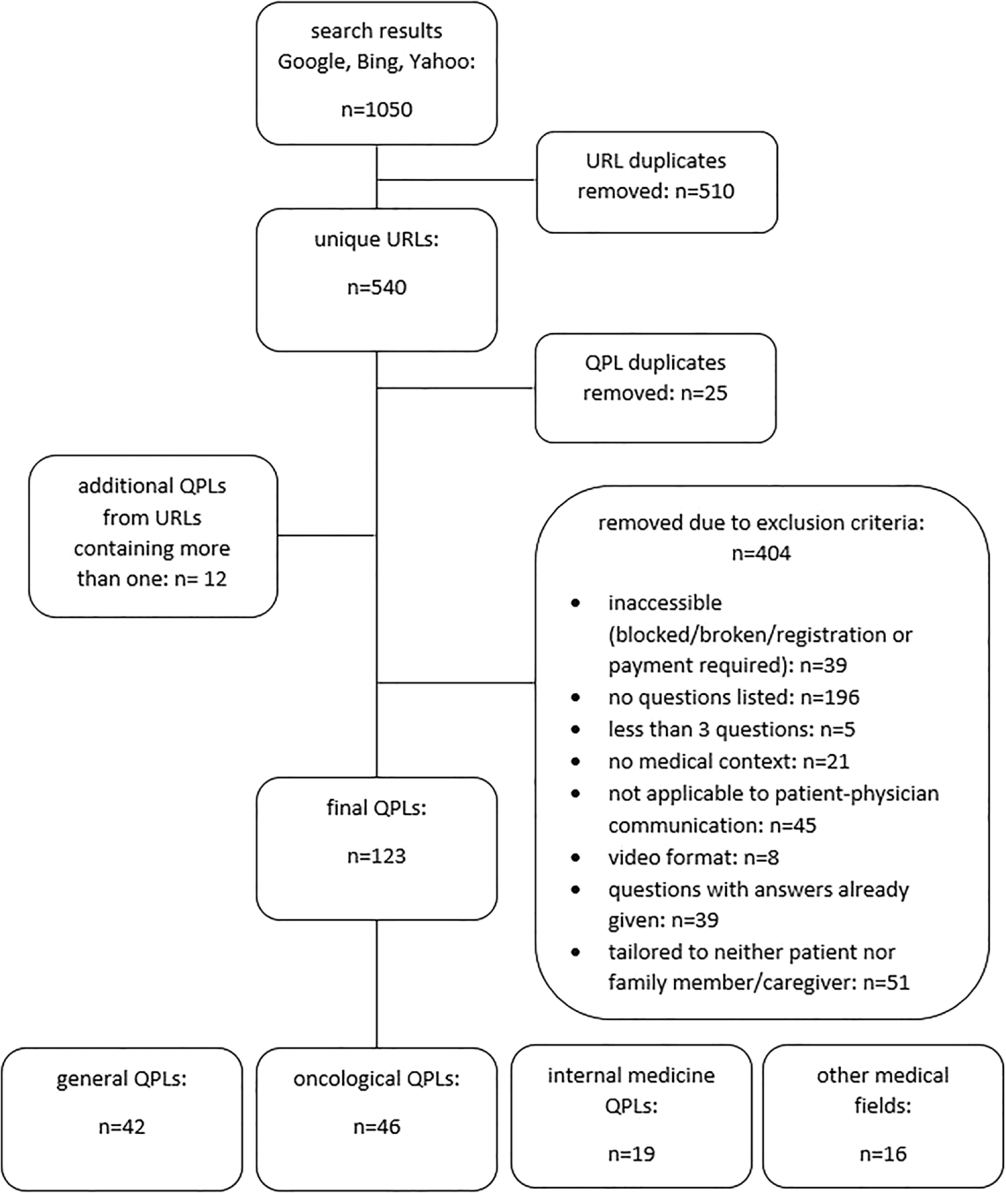
Flow chart of the online search for QPLs

The resulting QPLs were then sorted according to medical fields. The entire process is depicted in the flow chart in Fig. 1. This study focuses on the quality assessment of the resulting oncological QPLs.

### Quality Assessment

The tool we used to assess the QPLs’ quality is based on various established lists of quality criteria for different kinds of patient information material, such as the International Patient Decision Aid Standards (IPDAS), Health on the Net code of conduct (HONcode), and DISCERN [14–19]. In addition, we searched current literature for criteria used in studies developing or testing the effectiveness of QPLs. Criteria from all of those sources were extracted and checked for their fit for QPLs by three of the authors independently (JH, LJ, and NZ). Each of the three also made suggestions on which items to summarize due to their similarities. Results were compared, and differences were resolved through discussion until a set of preliminary criteria was achieved. This was used for a trial run with a selection of three different QPLs, and the raters’ feedback was used to further refine the items. The final criteria were then sorted into categories, and these categories were subsequently assigned to the two main groups of either content or formal criteria. Content criteria in this case refer to everything concerning the QPL’s content, which the user is to work with directly; formal criteria refer to everything concerning the QPL’s background, the development process, transparency, etc.

The resulting list of final criteria is given in an English translation in Table 1. For a complete list of all the resources and their extracted items that went into the development process, as well as the original German version of the final tool, see Online Resource 1.

**Table 1:** Items of the quality assessment tool sorted by category (English translation from the German original)

Category	Items
Goals^1^	• The goals of the publication are clearly stated • The target audience is clearly named • The QPL’s focus area(s) are easily identifiable
General contents^1^	• Contains instructions for use • Points out that users can assemble their own selection of questions/items • Instructions on how to draw up additional individual questions (half score (1 point only) if the option to create personal questions is only mentioned) • Items are complete (all relevant questions concerning the QPLs topic are addressed) • Avoids repetition/redundancy in items • Items have relevance for users of the target audience • Items are sorted in a reasonable, content-related way
Participating communication^1^	• Intended as a support and not a substitute for talking to the doctor (complementarity) • Explicitly encourages communication and guides the user through it step by step • Prompts users to clarify with the doctor whether there are different options and to discuss those • Clarifies that the formulations for the discussion with the doctor can be individually adapted and changed, and gives examples • Points out that there are several sources of information for the patient (beyond the doctor’s consultation)
Visualization^1^	• Neatly structured • Formatting (font type and size) is easy to read • No weighting of different items/topics by way of presentation • Images, if used within the text, support the content and acquisition/processing of information
Language^1^	• Precise wording of items • Unbiased wording of items • Comprehensible for all users of the target audience (language levels—assessed via formulas or field tests—attest comprehensibility; technical/medical terms are explained where necessary)
Quality management^2^	• Explains which steps were taken by the authors when selecting items • Date of creation is given • Information on whether QPL is up-to-date (date of the last update/information about the period of validity/frequency of updates/planned next update) • References to the involvement of experts as well as practice users (doctors and patients) in the development and review of the QPL
Transparency^2^	• Information about author(s) and publisher(s) • Contact details and feedback options • Disclosure about financing of development and distribution • Disclosure of potential profits for author(s)/publisher(s) • Clear separation between content and advertising (text/image)
Access^2^	• Low-threshold access for all patients within the target group at no additional cost • Offers other options than reading (e.g., audio, video, or in-person discussion) in order to prepare questions for the consultation with the doctor • Details about additional help and information
Online QPLs^2^	• Easy to find on the internet • Website facilitates return to QPL after following links to other websites • Website offers search for keyword • QPL can be printed as a single document (e.g., PDF) with the function embedded on the website
Online interactive QPLs^2^	• The website(s) facilitate navigation (e.g., back and forth not only via browser function) without losing previously entered information • Provides feedback on personal health information that can be included in the checklist • Security for any personal information provided • Explicit information on data use and protection

^1^Content-related criteria; ^2^formal criteria

Four independent raters (two physicians and two medical students) then used the tool to evaluate the 46 oncological QPLs. Raters were instructed to assign points from 0 to 2 for each item, where 0 meant that the item was not at all fulfilled by the QPL, 1 meant that the item was partly fulfilled, and 2 signified a completely satisfactory fulfillment. In case an item did not apply to a QPL (e.g., items referring to web-based versions of QPLs could not be applied to PDF documents), or in case an item could not be evaluated because the information was missing, the raters were instructed to indicate this instead of assigning points. Based on the item scores of 0–2, ratings for each QPL were first calculated for each rater separately in the form of mean values and percentages: (1) for every separate category, (2) for content and formal criteria, and (3) the overall result. Mean values and percentages for the overall rating were then also computed across all four raters. Inter-rater concordance was measured by Kendall’s coefficient of concordance [20], using the rankings of each rater’s overall score.

We tested for differences in quality assessment between QPLs designed by medical organizations, nonprofit organizations, and for-profit organizations using Kruskal–Wallis tests and Dunn–Bonferroni post hoc tests. The same methods were used to test for differences in quality assessment between general QPLs for cancer patients and those designed specifically for patients with the two most common types of cancer: breast and prostate cancer.

## Results

### Online Findings

The online search yielded 123 distinct QPLs. Forty-six of those specialized in oncological issues and were thus included in the quality assessment. Most of these checklists were generally focused on cancer diagnosis, treatment, and rehabilitation (22); of those for specific cancers, most focused on breast cancer (10), followed by prostate cancer (4), as determined by the additional, specified search terms (for a full list, see Online Resource 2).

More than half of the QPLs were published by pharmaceutical companies and other for-profit organizations (*n* = 29; 63%); the other half was divided between non-profit organizations (*n* = 7; 15.2%) and medical facilities (*n* = 10; 21.7%). The number of questions ranged between 5 and 147, with a mean of 16.5 questions (SD 27). All of them were directed at patients (two in more general terms), while none were specifically designed for caregivers, family members, etc.

### Quality Assessment

Kendall’s coefficient of concordance for the four raters in this study was 0.7 (*p* < 0.0001), pointing toward a reasonable level of agreement.

The range of the overall rating of all 46 evaluated QPLs lay between 21 and 40%. On average, the QPLs scored 27.4% (Mdn = 27.1%, SD = 4.4). Ratings differed considerably between the different subcategories. The highest-rated category was visualization (*M* = 90.8%), and the lowest was quality management (*M* = 15.4%). However, for all ten categories, there were QPLs achieving scores above 80%, with the exception of accessibility, whose maximum rating was 66.7%. Detailed results are presented in Table 2.

**Table 2:** Rating results by category

Category (*n* = 46, except where indicated)	Mean (%)	Median (%)	Standard deviation	Minimum (%)	Maximum (%)
*Goals*^**1**^	63.2	66.7	38.5	20.8	100
*General contents*^**1**^	57.1	53.6	13.2	39.3	92.9
*Participating communication*^**1**^	45.4	46.3	17.3	22.5	92.5
*Visualisation*^**1**^	90.8	95.8	13.1	43.8	100
*Language*^**1**^	87.6	87.5	9.1	66.7	100
*Quality management*^**2**^	15.4	12.5	16.7	0.0	84.4
*Transparency*^**2**^	42.7	45.0	17.7	7.5	85.0
*Access*^**2**^	44.2	44.4	11.7	22.2	66.7
*Online QPLs*^**2**^	49.3	43.8	20.0	16.7	87.5
*Online interactive QPLs*^**2**^* (n* = *1)*	87.5	87.5	/	87.5	87.5
*All content criteria*	64.5	65.2	11.0	50.0	90.3
*All formal criteria*	37.7	36.6	10.2	24.7	70.6
*Overall rating*	27.4	27.1	4.4	21.0	40.4

^1^Content-related criteria; ^2^formal criteria

### Qualitative Differences Between Publishers

**Fig. 2:**
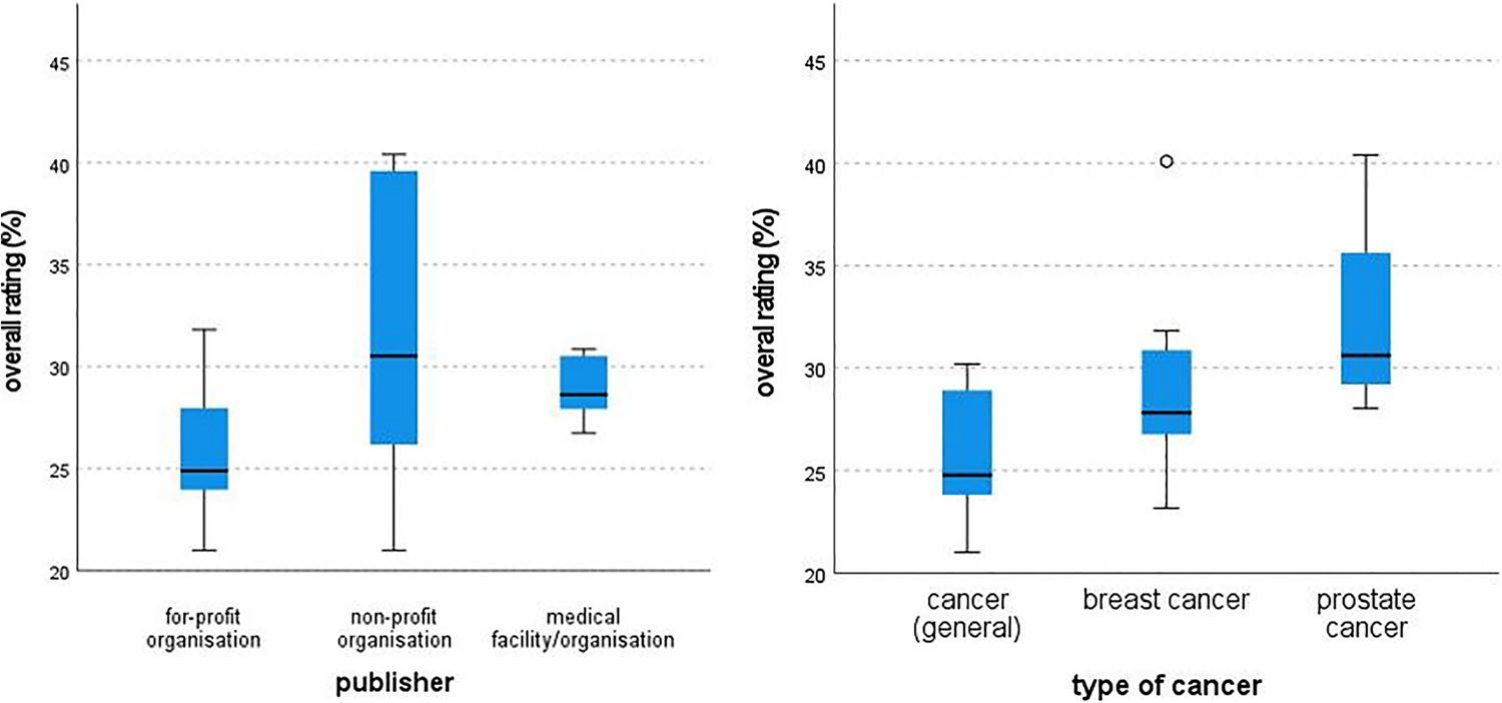
Differences in quality between publishers (left) and between QPLs for different types of cancer (right)

The results of the Kruskal–Wallis tests for differences in quality assessment between QPLs designed by medical organizations, non-profit organizations, and for-profit organizations showed a significantly higher overall quality of QPLs designed by medical organizations than of QPLs designed by for-profit organizations (*p* = 0.02; see Fig. 2). Regarding the quality of all content criteria, medical organizations, and non-profit organizations each show higher quality than for-profit organizations (*p* = 0.005 and *p* = 0.000 resp.). The quality of formal criteria did not differ significantly between any of the three groups.

Of all other subcategories, significant differences in quality were found for “goals,” “general contents,” and “participating communication.” Post hoc tests for those again revealed higher ratings for QPLs from medical organizations than from for-profit organizations (“goals”: *p* = 0.000), as well as higher ratings for QPLs from non-profit organizations compared to for-profit ones (“goals”: *p* = 0.002; “general contents”: *p* = 0.000; “participating communication” *p* = 0.001). Ratings of other subcategories did not differ significantly across the three groups of publishers.

### Qualitative Differences Between Types of Cancer

For the Kruskal–Wallis tests on differences in quality assessment between general QPLs for cancer patients and those designed specifically for breast and prostate cancer patients, group sizes differed considerably (general QPLs *n* = 22, breast cancer *n* = 10, and prostate cancer *n* = 4). Regarding the overall rating, specific QPLs for prostate cancer were of significantly higher quality than the general QPLs (*p* = 0.022; see Fig. 2). The content criteria were rated significantly better for both breast and prostate cancer QPLs than for general QPLs (*p* = 0.006 and *p* = 0.008, resp.), while no differences were found for the overall rating of formal criteria.

Among the subcategories, QPLs for breast cancer had significantly better ratings than general QPLs regarding “goals” (*p* = 0.000), “general content” (*p* = 0.004), “participating communication” (*p* = 0.017), and “visualization” (*p* = 0.001). QPLs for prostate cancer were also rated better in quality for the categories “goals” (*p* = 0.002) and “visualization” (*p* = 0.039).

## Discussion

The first noticeable result of this study is the generally low overall quality rating for German-language QPLs found online, the highest rating being just over 40%. As a recent systematic review [8] found, patients have a tendency to perceive QPLs as more helpful than information sheets, highlighting the need for good quality QPLs to be made available for cancer patients. From the results of this study, we must assume that either there are no QPLs of better quality for German-speaking oncological patients or that they are difficult to access and thus did not make it into the pool for our final evaluation.

The high maximum ratings of the individual subcategories (all above 80% with one exception) demonstrate that high values can be achieved in principle for every category. In light of this, we can assume that the quality criteria we collected for the assessment tool in this study are reasonable and have been considered and largely adhered to by authors of QPLs at some point. Nevertheless, overall ratings are low, as no QPL achieves high to average ratings in the majority of categories. This result leads to the assumption, that during the construction of QPLs, only a fairly limited number of quality aspects tend to be considered.

The result that the “visualization” category received the best rating may be due to the content of QPLs being relatively easy to structure. Not many images are needed in order to explain complicated aspects; thus, the demands placed on this category are not very high when evaluating QPLs. The finding that “access” never achieves truly satisfactory ratings highlights a problem that is evident with lots of different types of patient information: Any groups of patients struggling with ordinary reading (due to blindness, dyslexia, illiteracy, migration, etc.) are frequently underrepresented. The QPLs found in this study also rarely considered this issue; only one out of 46 linked to an additional video giving simple explanations on how to prepare for a medical consultation.

While the overall quality of QPLs published by for-profit organizations is lower than those published by medical organizations and facilities (and regarding content only also lower than of those published by non-profit organizations), the number of QPLS from for-profit organizations we found is much larger. One conclusion to draw from this may be that medical organizations need to invest in producing more material for patients. Another reason for this comparatively large number of QPLs from for-profit organizations may be that the websites of such organizations are easier to find and more visible in online searches [21, 22].

When creating QPLs, focusing on specific patient groups may make it easier to meet quality criteria, as demonstrated by the differences between specific breast and prostate cancer QPLs on the one hand and general cancer QPLs on the other. These findings need to be interpreted with caution, however, due to the considerable differences in group size. An alternative explanation for this difference in quality may be derived from the fact that especially breast cancer is often the focus of studies that examine adjuvant and complementary therapies, patient needs, psychological aspects in oncology, etc. As a result, both physicians and patients are potentially better educated regarding communication and the use of supporting materials and patient information, and their demand for such material is higher, while there is also more evidence already to help produce good-quality material. This would corroborate the earlier conclusion that, with enough effort, focus, and the appropriate scientific basis, high-quality QPLs can be produced that meet the criteria proposed in this study.

The ambiguous results of effectiveness studies and meta-analyses regarding QPLs [7–10] may be explained at least in part by the large variation in the quality of the available material. We were unable to find meta-analyses on the effectiveness of QPLs that took into account this large variety in quality, structure, or origin of the evaluated QPLs—often because the original studies themselves are sparse on explicit information of the QPLs being used.

Since our study was limited to German-language QPLs, the question arises whether the findings also need to be limited to material produced in German. Some of our findings may be a direct or indirect consequence of how the German healthcare system is organized. Others may result from the still very limited research on the decisive aspects that make QPLs effective, and they may appear similar in other languages and cultures. Looking at international research, we found one systematic review that tried to assess the general methodological quality of QPLs along with their effectiveness [7]. It suggests that the characteristics of a QPL intervention influence its effectiveness, but the study did not examine these characteristics further. We recommend that similar examinations be carried out in other languages and cultures to help better understand what makes the increasingly popular tool of a QPL effective and where the presently existing material needs to be improved to better support patients and physicians in their communicative challenges.

### Limitations

There are several aspects of this study—aside from the self-imposed limitations (German language only, three search engines, a maximum of 50 results per search)—that limit the interpretations of its results. The terms used to search for QPLs were derived from scientific literature, and the material from a preliminary search. In order to reproduce more accurate results of what oncological patients—who usually start out as laypeople in this field—would find, search terms should be generated by assessing the patients’ de facto approaches.

The assessment tool used to evaluate the QPLs was newly developed and not yet tested on a larger group of raters. While the tool was built drawing upon internationally accepted standards and the inter-rater concordance in this study seems reasonable enough, the tool has yet to be evaluated, particularly regarding its use by people with no medical background or expertise. It already became evident that some items of the tool might need further specifications and instructions for raters, e.g., reference points for optimal font size and type for specific user groups based on scientific data.

Furthermore, no additional analysis on visibility of the different websites containing QPLs was conducted. This might have shed further light on why there were so many more QPLs from for-profit organizations than from non-profit/medical organizations.

## Conclusion

The QPLs currently available online to German-speaking oncological patients leave a lot to be desired. The findings of this study demonstrate that higher quality can be achieved in all categories considered. Therefore, as long as there are reasonable grounds to assume QPLs are a valuable support to help patients cope by better understanding their diagnosis and treatment, more efforts should be made to provide them with good-quality material—by preselecting existing QPLs or creating new ones.

Regarding future research, quality criteria for the QPLs themselves need to be taken into account when studying their efficacy and effectiveness in everyday clinical practice. In validating quality assessment tools, potential differences between raters with varying expertise should be examined further, and lay people’s perspectives should be added as well.

In conclusion, the quality criteria presented in this study constitute a useful tool when (a) trying to find material that is safe to recommend and to be used by patients, (b) creating new QPLs to support patient-physician communication, and (c) studying the effectiveness of QPL interventions.

### Supplementary Information

Below is the link to the electronic supplementary material.

Supplementary file1 (XLSX 51 KB)

Supplementary file2 (DOCX 16 KB)
