# Supplementary material for: Development and Application of a Quality Assessment Tool for Oncological Question Prompt Lists
Source: J Cancer Educ. 2023 Mar 30;38(5):1493–500. doi: 10.1007/s13187-023-02290-z (PMC10509062; doi:10.1007/s13187-023-02290-z)
Supplement: Supplementary file 2 — Supplementary file2 (DOCX 16 KB) [file 13187_2023_2290_MOESM2_ESM.docx]

| **Publisher** | **URL** |
| --- | --- |
| Deutsches Krebsforschungszentrum & Krebsinformationsdienst | <https://www.krebsinformationsdienst.de/service/iblatt/iblatt-behandlungswahl.pdf> |
| Bild GmbH | <https://www.bild.de/ratgeber/gesundheit/brustkrebs/darauf-sollte-ich-achten-51699968.bild.html> |
| Advigon Versicherung AG | <https://www.advigon.com/de/ratgeber/krebsratgeber/checkliste-fuer-die-ambulante-krebstherapie> |
| Selbsthilfegruppe Pankreaskarzinom | <http://www.selbsthilfe-pankreaskarzinom.at/service/checkliste-fuer-den-arztbesuch.html> |
| Bristol Meyers Squibb | <https://www.fl-haematologie.info/ratgeber/infothek/checklisten-fuer-das-arztgespraech/> |
| Dr. med. Iris Herscovici | <https://selpers.com/tools/fragen-an-den-arzt-bei-brustkrebs/> |
| AstraZeneca / MSD SHARP & DOHME GMBH | <https://www.wegweiser-eierstockkrebs.de/welche-fragen-sollte-ich-dem-arzt-stellen> |
| Onkologische Schwerpunktpraxis Tiergarten Berlin | <https://www.onkologie-tiergarten.de/informationen/fragen_an_meinen_arzt/> |
| Deutsches Krebsforschungszentrum & Krebsinformationsdienst | <https://www.krebsinformationsdienst.de/service/iblatt/prostatakrebs-lokal-flyer.pdf?m=1573033516&> |
| Felix Burda Stiftung München | <https://www.darmkrebs.de/fragen-an-ihren-arzt> |
| Dr. med. Iris Herscovici | <https://selpers.com/tools/fragen-an-den-arzt-bei-lymphomen/> |
| GFMK GmbH & Co. KG | <https://www.curado.de/fragen-arzt-32041> |
| Austrian Breast and Colorectal Cancer Study Group (ABCSG) | <https://www.brustkrebsexperten.at/informationen-fuer-patientinnen-und-patienten/fragen-an-den-arzt-beispiel-fuer-einen-fragenkatalog/> |
| Bundesverband Schilddrüsenkrebs Ohne Schilddrüse leben e.V. | <https://www.sd-krebs.de/pdf/Merkblatt_Gespraech_20140615.pdf> |
| Roche Pharma AG | <https://daskwort.de/unterstuetzung/broschueren-und-infomaterialien/checkliste-diagnose-krebs> |
| Pfizer Pharma GmbH | <https://www.esgehtummich-brustkrebs.de/behandlung-metastasierter-brustkrebs#vorbereitung-auf-das-arztgespraech> |
| Roche Pharma AG | <https://daskwort.de/krebsarten/brustkrebs/behandlungsmoeglichkeiten-bei-metastasiertem-brustkrebs/> |
| Astellas Pharma GmbH | <https://www.meine-prostata.de/vorsorge/vorsorgeservice#vorbereitung_arztgespraech> |
| Dr. med. Iris Herscovici | <https://selpers.com/tools/fragen-an-den-arzt-bei-prostatakrebs/> |
| Roche Pharma AG | <https://daskwort.de/unterstuetzung/broschueren-und-infomaterialien/checkliste-nach-der-krebstherapie> |
| Roche Pharma AG | <https://daskwort.de/unterstuetzung/broschueren-und-infomaterialien/checkliste-reha-bei-krebs> |
| Roche Pharma AG | <https://daskwort.de/unterstuetzung/broschueren-und-infomaterialien/checkliste-behandlung> |
| Roche Pharma AG | <https://daskwort.de/unterstuetzung/broschueren-und-infomaterialien/checkliste-komplementaermedizin-bei-krebs> |
| Bristol Meyers Squibb | <https://www.krebs.de/servlet/servlet.FileDownload?file=00P0J00001WotNhUAJ> |
| Bristol Meyers Squibb | <https://www.krebs.de/servlet/servlet.FileDownload?file=00P0J00001WotPNUAZ> |
| AstraZeneca GmbH | <https://teste-deinen-lungenkrebs.de/#check> |
| Institut für Medizinsoziologie, Versorgungsforschung und Rehabilitationswissenschaft der Universität zu Köln (IMVR) | <https://www.frauenselbsthilfe.de/_Resources/Persistent/b8f27b967bcc4139f65c0d65264260481bbbdc53/2018-05-22-Hilfe%20bei%20Brustkrebs.pdf> |
| Pfizer Pharma GmbH | <https://www.hilfefuermich.de/wie-kann-ich-mich-am-besten-auf-das-arztgespraech-vorbereiten-1> |
| Arbeitsgemeinschaft Deutscher Darmkrebszentren e. V. (ADDZ) | <https://www.ag-darmzentren.com/darmkrebs-information/fragen-an-den-arzt> |
| Dr. med. Iris Herscovici | <https://selpers.com/tools/fragen-an-den-arzt-bei-lungenkrebs/> |
| Berliner Ärzte-Verlag GmbH | <http://www.brustkrebs-web.de/kontrakrebs/843_fragen.php> |
| Amoena Medizin-Orthopädie-Technik GmbH | <https://www.amoena.com/de/Documents/Editor/Amoena-Broschuere-Mastektomie-DE_ePaper.pdf> |
| „Leitlinienprogramm Onkologie“ der Arbeitsgemeinschaft der Wis- senschaftlichen Medizinischen Fachgesellschaften e. V., der Deut- schen Krebsgesellschaft e. V. und der Deutschen Krebshilfe e. V. | <https://www.florence-nightingale-krankenhaus.de/fileadmin/daten/fnk/Klinik-fuer-Geburtshilfe-und-Gynaekologie/Dateien/Patientenleitlinie_Brustkrebs1.pdf> |
| „Leitlinienprogramm Onkologie“ der Arbeitsgemeinschaft der Wissenschaftlichen Medizinischen Fachgesellschaften e. V., der Deutschen Krebsgesellschaft e. V. und der Deutschen Krebshilfe e. V. | <https://www.urologenportal.de/fileadmin/MDB/PDF/PCA_1.pdf> |
| Merck Gesellschaft mbH | <https://diagnose-krebs.at/wp-content/uploads/2020/06/fragebogen_01.pdf> |
| Merck Gesellschaft mbH | <https://diagnose-krebs.at/wp-content/uploads/2020/06/fragebogen_02.pdf> |
| Merck Gesellschaft mbH | <https://diagnose-krebs.at/wp-content/uploads/2020/06/fragebogen_03.pdf> |
| Merck Gesellschaft mbH | <https://diagnose-krebs.at/wp-content/uploads/2020/06/fragebogen_04.pdf> |
| Merck Gesellschaft mbH | <https://diagnose-krebs.at/wp-content/uploads/2020/06/fragebogen_05.pdf> |
| Merck Gesellschaft mbH | <https://diagnose-krebs.at/wp-content/uploads/2020/06/fragebogen_06.pdf> |
| Merck Gesellschaft mbH | <https://diagnose-krebs.at/wp-content/uploads/2020/06/fragebogen_07.pdf> |
| Merck Gesellschaft mbH | <https://diagnose-krebs.at/wp-content/uploads/2020/06/fragebogen_08.pdf> |
| Merck Gesellschaft mbH | <https://diagnose-krebs.at/wp-content/uploads/2020/06/fragebogen_09.pdf> |
| Merck Gesellschaft mbH | <https://diagnose-krebs.at/wp-content/uploads/2020/06/fragebogen_10.pdf> |
| Merck Gesellschaft mbH | <https://diagnose-krebs.at/wp-content/uploads/2020/06/fragebogen_11.pdf> |
| Merck Gesellschaft mbH | <https://diagnose-krebs.at/wp-content/uploads/2020/06/fragebogen_12.pdf> |
